# Supplementary figures and images for: Adaptation and compensation in a bacterial gene regulatory network evolving under antibiotic selection
Source: eLife. 2021 Sep 30;10:e70931. doi: 10.7554/eLife.70931 (PMC8483737; doi:10.7554/eLife.70931)

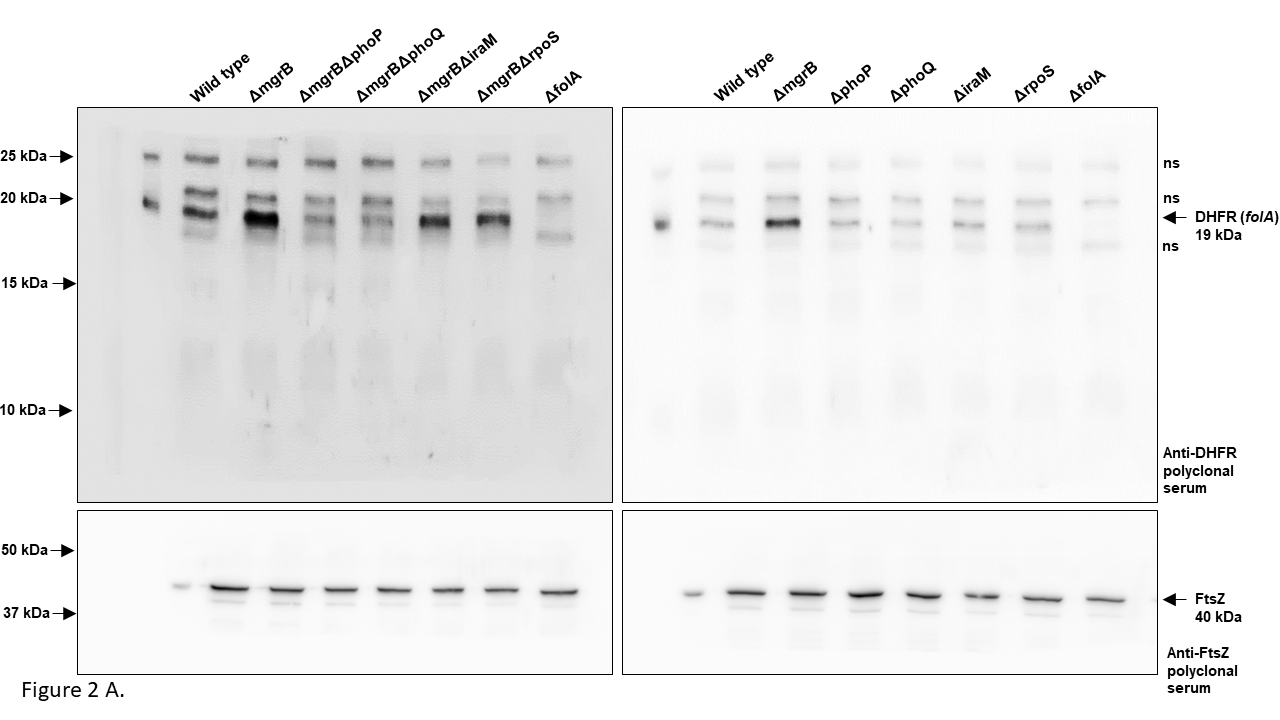

Supplement: Source data 1. — Figure 2. Uncropped, annotated immunoblot (Source data 1) and raw image files (Source data 2, 3) for DHFR and FtsZ in various E. coli mutants. Specific bands were identified based on molecular weight and absence from lysate from E. coli ΔfolA. Figure 2D. Uncropped, annotated immunoblot (Source data 1) and raw image files (Source data 2, 3, 4) for DHFR and FtsZ in various trimethoprim resistant E. coli mutants. Specific bands were identified based on molecular weight and absence from lysate from E. coli ΔfolA. Figure 2E. Uncropped, annotated immunoblot (Source data 1) and raw image files (Source data 2, 3, 4) for DHFR and FtsZ in various trimethoprim resistant E. coli mutants (TMPR1-5) and their ΔphoP derivatives. Specific bands were identified based on molecular weight and absence from lysate from E. coli ΔfolA. Figure 3E. Uncropped, annotated immunoblot (Source data 1) and raw image files (Source data 2, 3) for plasmid-expressed His-tagged DHFR or its mutant alleles in E. coli in the presence of indicated concentrations of inducer (IPTG). Figure 2—figure supplement 3. Uncropped, annotated immunoblot (Source data 1) and raw image files (Source data 2, 3) for DHFR and FtsZ in indicated E. coli mutants. Specific bands were identified based on molecular weight and absence from lysate from E. coli ΔfolA. [file elife-70931-supp4.zip › Source data-revised/Figure 2A-source data 1.tif]

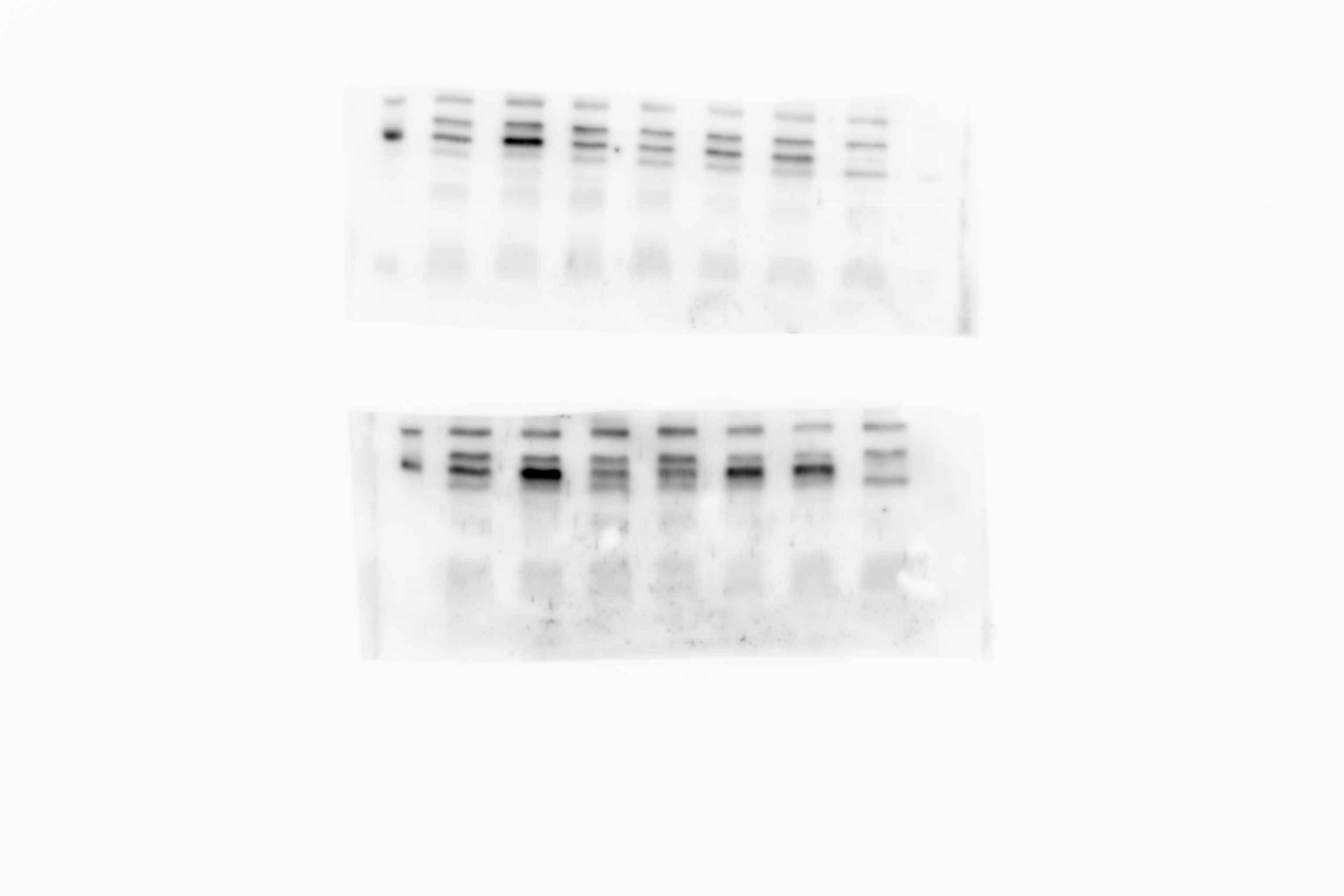

Supplement: Source data 1. — Figure 2. Uncropped, annotated immunoblot (Source data 1) and raw image files (Source data 2, 3) for DHFR and FtsZ in various E. coli mutants. Specific bands were identified based on molecular weight and absence from lysate from E. coli ΔfolA. Figure 2D. Uncropped, annotated immunoblot (Source data 1) and raw image files (Source data 2, 3, 4) for DHFR and FtsZ in various trimethoprim resistant E. coli mutants. Specific bands were identified based on molecular weight and absence from lysate from E. coli ΔfolA. Figure 2E. Uncropped, annotated immunoblot (Source data 1) and raw image files (Source data 2, 3, 4) for DHFR and FtsZ in various trimethoprim resistant E. coli mutants (TMPR1-5) and their ΔphoP derivatives. Specific bands were identified based on molecular weight and absence from lysate from E. coli ΔfolA. Figure 3E. Uncropped, annotated immunoblot (Source data 1) and raw image files (Source data 2, 3) for plasmid-expressed His-tagged DHFR or its mutant alleles in E. coli in the presence of indicated concentrations of inducer (IPTG). Figure 2—figure supplement 3. Uncropped, annotated immunoblot (Source data 1) and raw image files (Source data 2, 3) for DHFR and FtsZ in indicated E. coli mutants. Specific bands were identified based on molecular weight and absence from lysate from E. coli ΔfolA. [file elife-70931-supp4.zip › Source data-revised/Figure 2A-source data 2.tif]

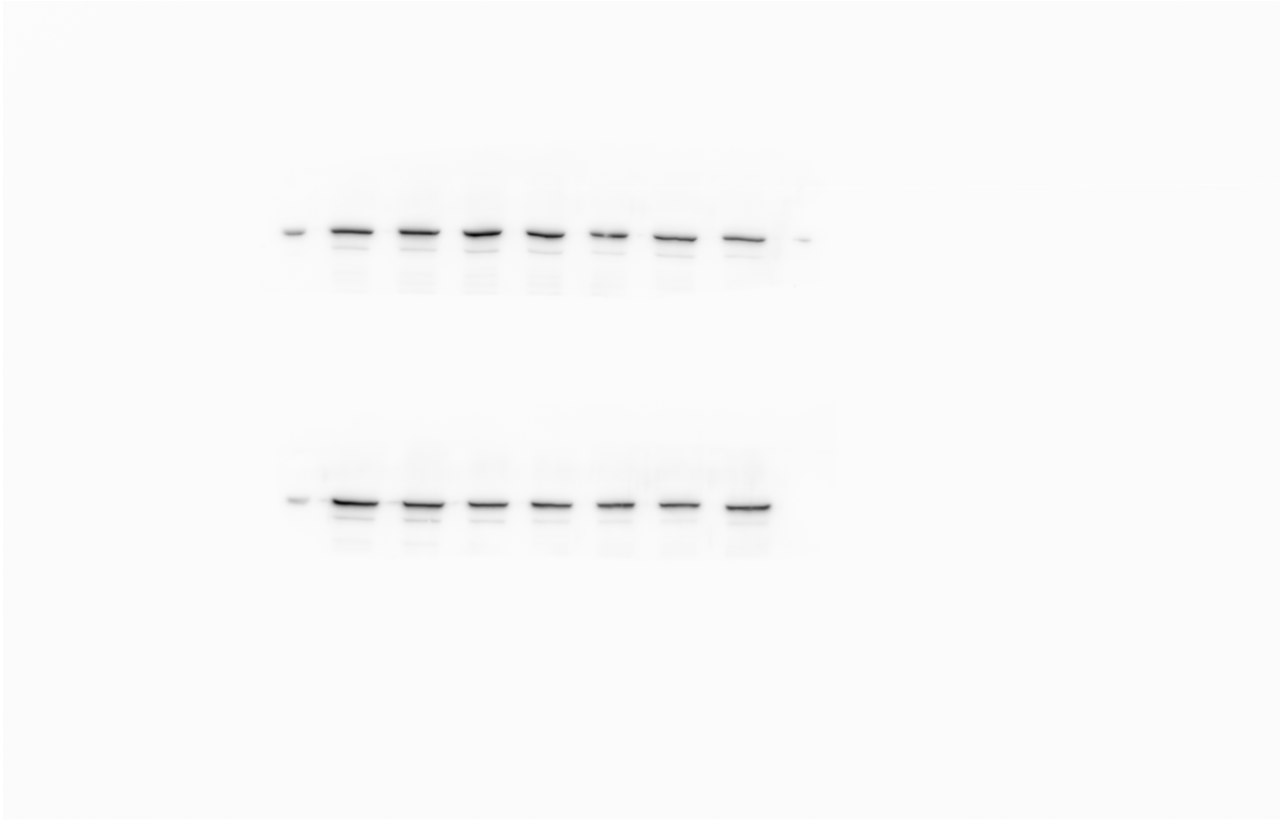

Supplement: Source data 1. — Figure 2. Uncropped, annotated immunoblot (Source data 1) and raw image files (Source data 2, 3) for DHFR and FtsZ in various E. coli mutants. Specific bands were identified based on molecular weight and absence from lysate from E. coli ΔfolA. Figure 2D. Uncropped, annotated immunoblot (Source data 1) and raw image files (Source data 2, 3, 4) for DHFR and FtsZ in various trimethoprim resistant E. coli mutants. Specific bands were identified based on molecular weight and absence from lysate from E. coli ΔfolA. Figure 2E. Uncropped, annotated immunoblot (Source data 1) and raw image files (Source data 2, 3, 4) for DHFR and FtsZ in various trimethoprim resistant E. coli mutants (TMPR1-5) and their ΔphoP derivatives. Specific bands were identified based on molecular weight and absence from lysate from E. coli ΔfolA. Figure 3E. Uncropped, annotated immunoblot (Source data 1) and raw image files (Source data 2, 3) for plasmid-expressed His-tagged DHFR or its mutant alleles in E. coli in the presence of indicated concentrations of inducer (IPTG). Figure 2—figure supplement 3. Uncropped, annotated immunoblot (Source data 1) and raw image files (Source data 2, 3) for DHFR and FtsZ in indicated E. coli mutants. Specific bands were identified based on molecular weight and absence from lysate from E. coli ΔfolA. [file elife-70931-supp4.zip › Source data-revised/Figure 2A-source data 3.tif]

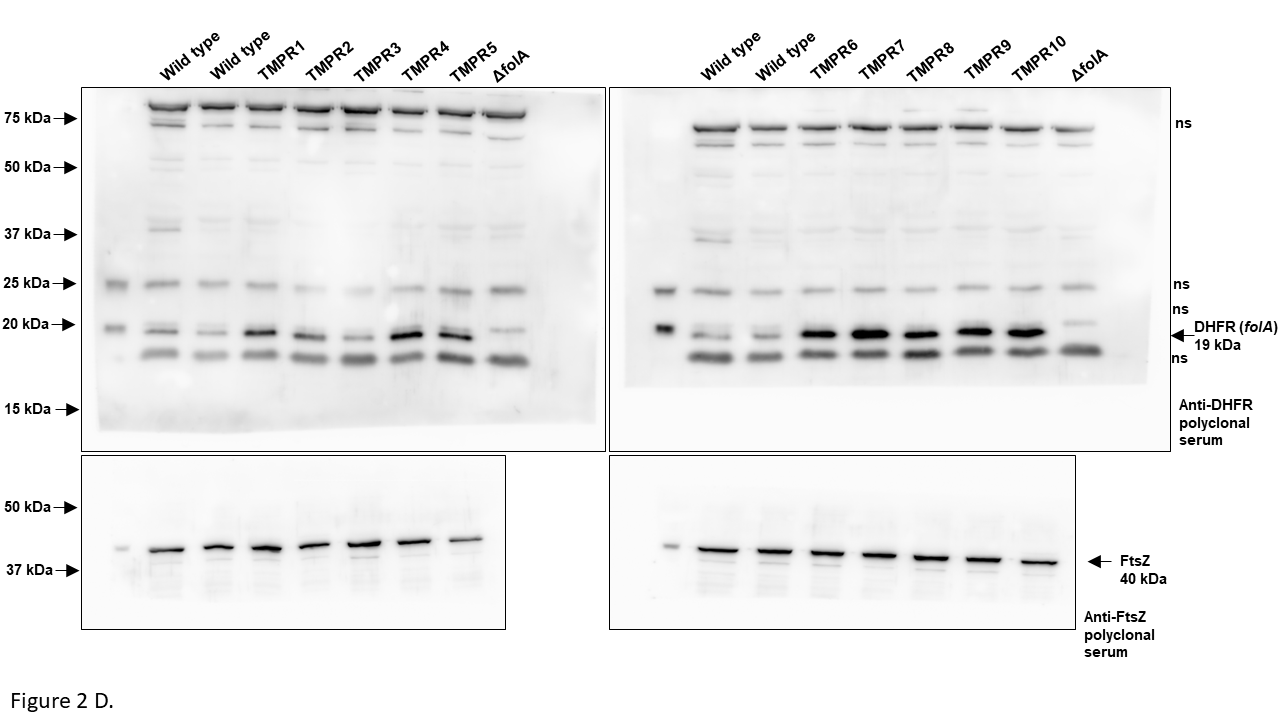

Supplement: Source data 1. — Figure 2. Uncropped, annotated immunoblot (Source data 1) and raw image files (Source data 2, 3) for DHFR and FtsZ in various E. coli mutants. Specific bands were identified based on molecular weight and absence from lysate from E. coli ΔfolA. Figure 2D. Uncropped, annotated immunoblot (Source data 1) and raw image files (Source data 2, 3, 4) for DHFR and FtsZ in various trimethoprim resistant E. coli mutants. Specific bands were identified based on molecular weight and absence from lysate from E. coli ΔfolA. Figure 2E. Uncropped, annotated immunoblot (Source data 1) and raw image files (Source data 2, 3, 4) for DHFR and FtsZ in various trimethoprim resistant E. coli mutants (TMPR1-5) and their ΔphoP derivatives. Specific bands were identified based on molecular weight and absence from lysate from E. coli ΔfolA. Figure 3E. Uncropped, annotated immunoblot (Source data 1) and raw image files (Source data 2, 3) for plasmid-expressed His-tagged DHFR or its mutant alleles in E. coli in the presence of indicated concentrations of inducer (IPTG). Figure 2—figure supplement 3. Uncropped, annotated immunoblot (Source data 1) and raw image files (Source data 2, 3) for DHFR and FtsZ in indicated E. coli mutants. Specific bands were identified based on molecular weight and absence from lysate from E. coli ΔfolA. [file elife-70931-supp4.zip › Source data-revised/Figure 2D-source data 1.tif]

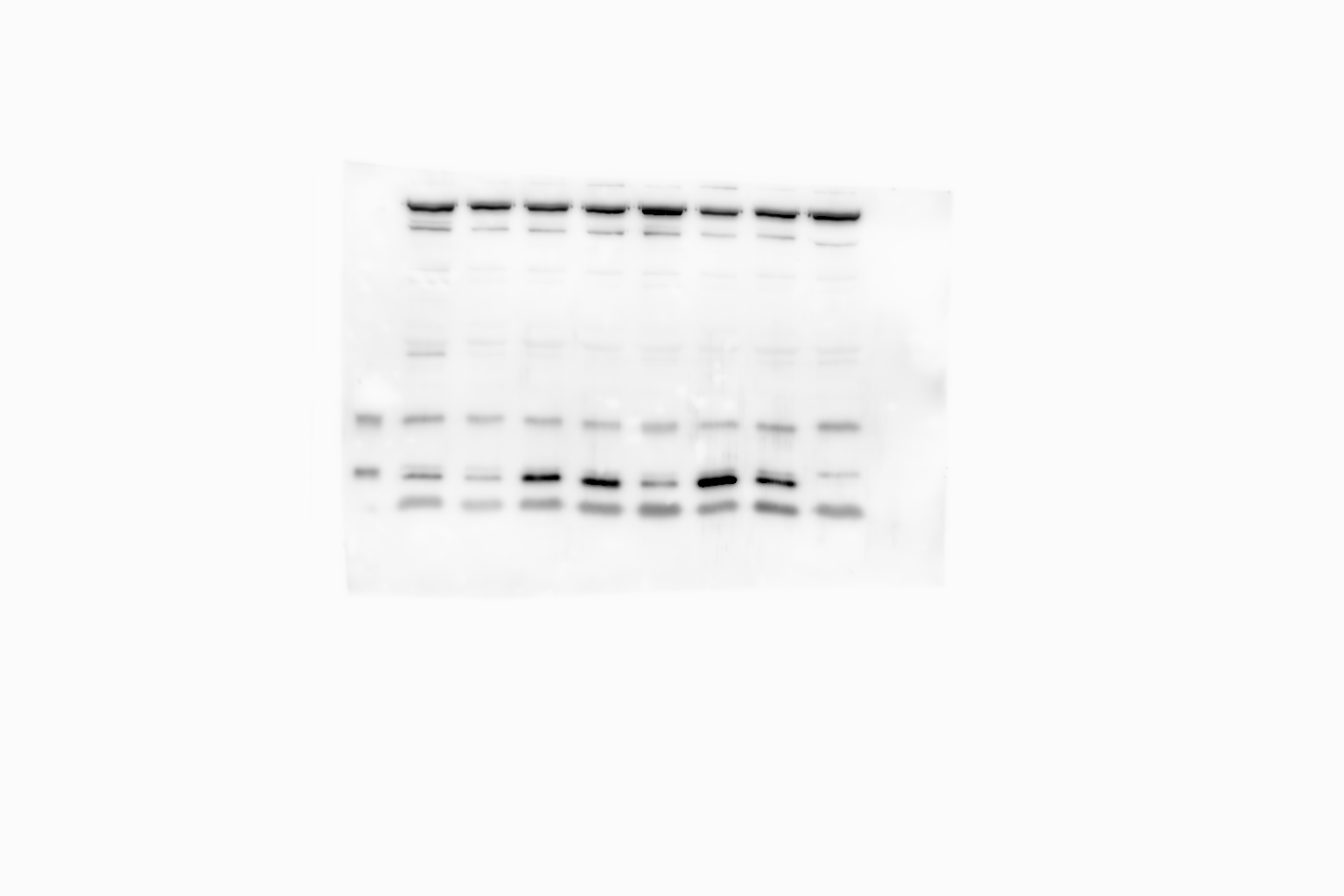

Supplement: Source data 1. — Figure 2. Uncropped, annotated immunoblot (Source data 1) and raw image files (Source data 2, 3) for DHFR and FtsZ in various E. coli mutants. Specific bands were identified based on molecular weight and absence from lysate from E. coli ΔfolA. Figure 2D. Uncropped, annotated immunoblot (Source data 1) and raw image files (Source data 2, 3, 4) for DHFR and FtsZ in various trimethoprim resistant E. coli mutants. Specific bands were identified based on molecular weight and absence from lysate from E. coli ΔfolA. Figure 2E. Uncropped, annotated immunoblot (Source data 1) and raw image files (Source data 2, 3, 4) for DHFR and FtsZ in various trimethoprim resistant E. coli mutants (TMPR1-5) and their ΔphoP derivatives. Specific bands were identified based on molecular weight and absence from lysate from E. coli ΔfolA. Figure 3E. Uncropped, annotated immunoblot (Source data 1) and raw image files (Source data 2, 3) for plasmid-expressed His-tagged DHFR or its mutant alleles in E. coli in the presence of indicated concentrations of inducer (IPTG). Figure 2—figure supplement 3. Uncropped, annotated immunoblot (Source data 1) and raw image files (Source data 2, 3) for DHFR and FtsZ in indicated E. coli mutants. Specific bands were identified based on molecular weight and absence from lysate from E. coli ΔfolA. [file elife-70931-supp4.zip › Source data-revised/Figure 2D-source data 2.tif]

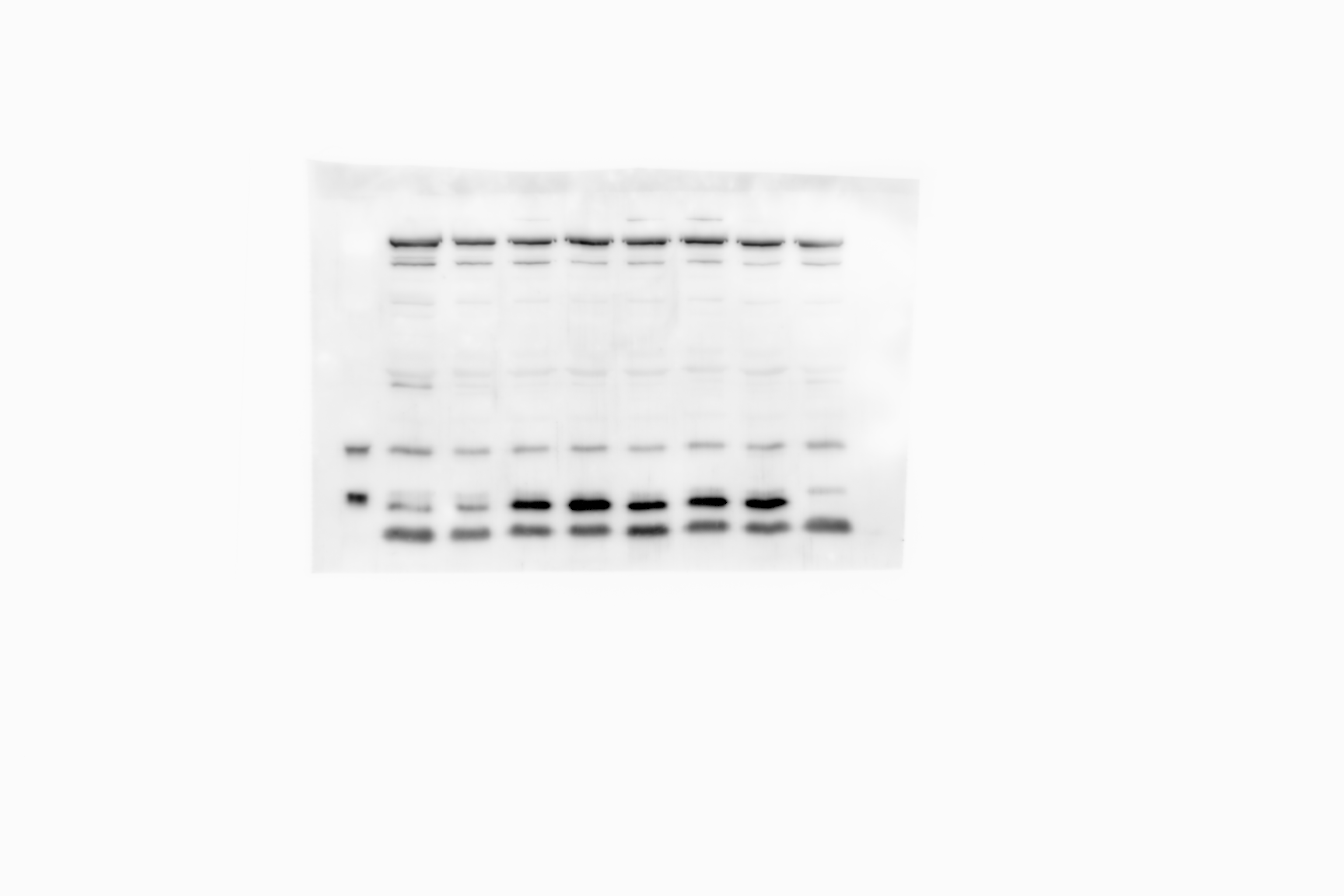

Supplement: Source data 1. — Figure 2. Uncropped, annotated immunoblot (Source data 1) and raw image files (Source data 2, 3) for DHFR and FtsZ in various E. coli mutants. Specific bands were identified based on molecular weight and absence from lysate from E. coli ΔfolA. Figure 2D. Uncropped, annotated immunoblot (Source data 1) and raw image files (Source data 2, 3, 4) for DHFR and FtsZ in various trimethoprim resistant E. coli mutants. Specific bands were identified based on molecular weight and absence from lysate from E. coli ΔfolA. Figure 2E. Uncropped, annotated immunoblot (Source data 1) and raw image files (Source data 2, 3, 4) for DHFR and FtsZ in various trimethoprim resistant E. coli mutants (TMPR1-5) and their ΔphoP derivatives. Specific bands were identified based on molecular weight and absence from lysate from E. coli ΔfolA. Figure 3E. Uncropped, annotated immunoblot (Source data 1) and raw image files (Source data 2, 3) for plasmid-expressed His-tagged DHFR or its mutant alleles in E. coli in the presence of indicated concentrations of inducer (IPTG). Figure 2—figure supplement 3. Uncropped, annotated immunoblot (Source data 1) and raw image files (Source data 2, 3) for DHFR and FtsZ in indicated E. coli mutants. Specific bands were identified based on molecular weight and absence from lysate from E. coli ΔfolA. [file elife-70931-supp4.zip › Source data-revised/Figure 2D-source data 3.tif]

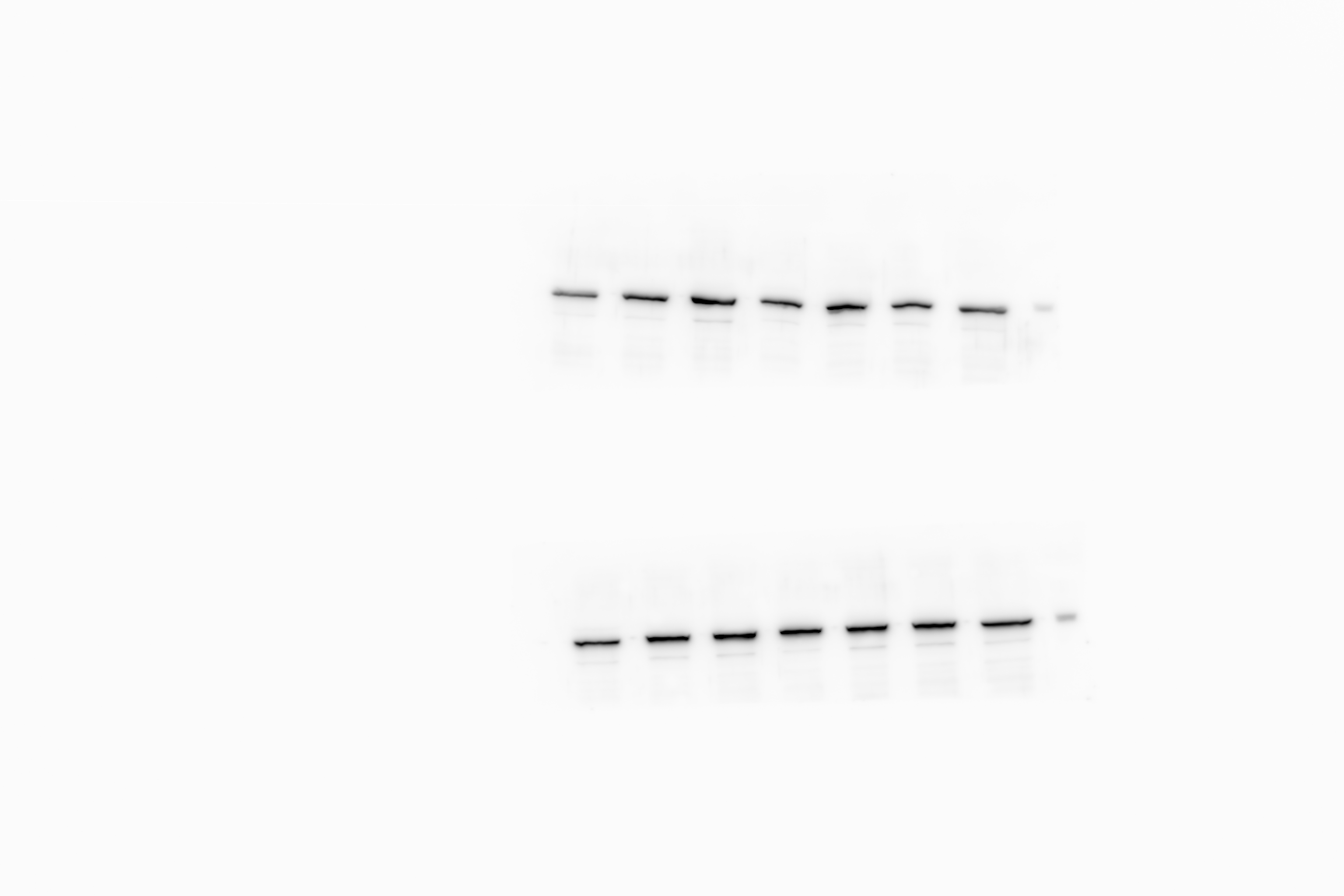

Supplement: Source data 1. — Figure 2. Uncropped, annotated immunoblot (Source data 1) and raw image files (Source data 2, 3) for DHFR and FtsZ in various E. coli mutants. Specific bands were identified based on molecular weight and absence from lysate from E. coli ΔfolA. Figure 2D. Uncropped, annotated immunoblot (Source data 1) and raw image files (Source data 2, 3, 4) for DHFR and FtsZ in various trimethoprim resistant E. coli mutants. Specific bands were identified based on molecular weight and absence from lysate from E. coli ΔfolA. Figure 2E. Uncropped, annotated immunoblot (Source data 1) and raw image files (Source data 2, 3, 4) for DHFR and FtsZ in various trimethoprim resistant E. coli mutants (TMPR1-5) and their ΔphoP derivatives. Specific bands were identified based on molecular weight and absence from lysate from E. coli ΔfolA. Figure 3E. Uncropped, annotated immunoblot (Source data 1) and raw image files (Source data 2, 3) for plasmid-expressed His-tagged DHFR or its mutant alleles in E. coli in the presence of indicated concentrations of inducer (IPTG). Figure 2—figure supplement 3. Uncropped, annotated immunoblot (Source data 1) and raw image files (Source data 2, 3) for DHFR and FtsZ in indicated E. coli mutants. Specific bands were identified based on molecular weight and absence from lysate from E. coli ΔfolA. [file elife-70931-supp4.zip › Source data-revised/Figure 2D-source data 4.tif]

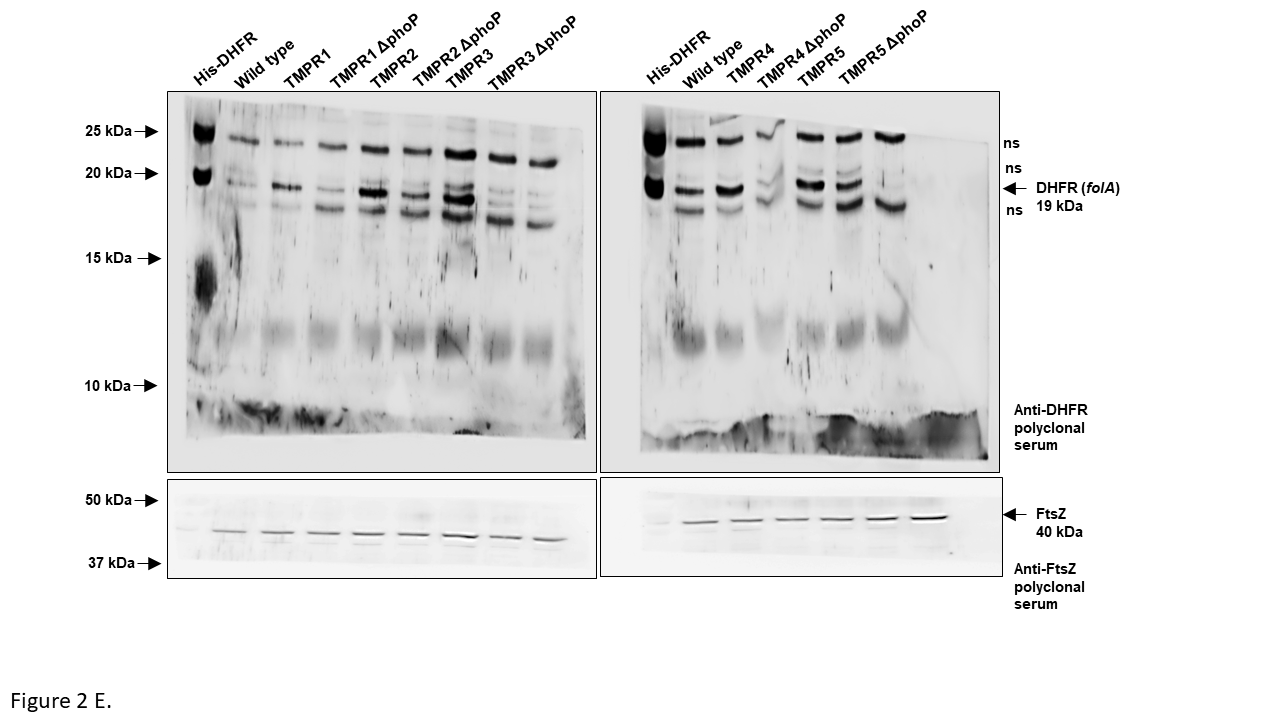

Supplement: Source data 1. — Figure 2. Uncropped, annotated immunoblot (Source data 1) and raw image files (Source data 2, 3) for DHFR and FtsZ in various E. coli mutants. Specific bands were identified based on molecular weight and absence from lysate from E. coli ΔfolA. Figure 2D. Uncropped, annotated immunoblot (Source data 1) and raw image files (Source data 2, 3, 4) for DHFR and FtsZ in various trimethoprim resistant E. coli mutants. Specific bands were identified based on molecular weight and absence from lysate from E. coli ΔfolA. Figure 2E. Uncropped, annotated immunoblot (Source data 1) and raw image files (Source data 2, 3, 4) for DHFR and FtsZ in various trimethoprim resistant E. coli mutants (TMPR1-5) and their ΔphoP derivatives. Specific bands were identified based on molecular weight and absence from lysate from E. coli ΔfolA. Figure 3E. Uncropped, annotated immunoblot (Source data 1) and raw image files (Source data 2, 3) for plasmid-expressed His-tagged DHFR or its mutant alleles in E. coli in the presence of indicated concentrations of inducer (IPTG). Figure 2—figure supplement 3. Uncropped, annotated immunoblot (Source data 1) and raw image files (Source data 2, 3) for DHFR and FtsZ in indicated E. coli mutants. Specific bands were identified based on molecular weight and absence from lysate from E. coli ΔfolA. [file elife-70931-supp4.zip › Source data-revised/Figure 2E-source data 1.tif]

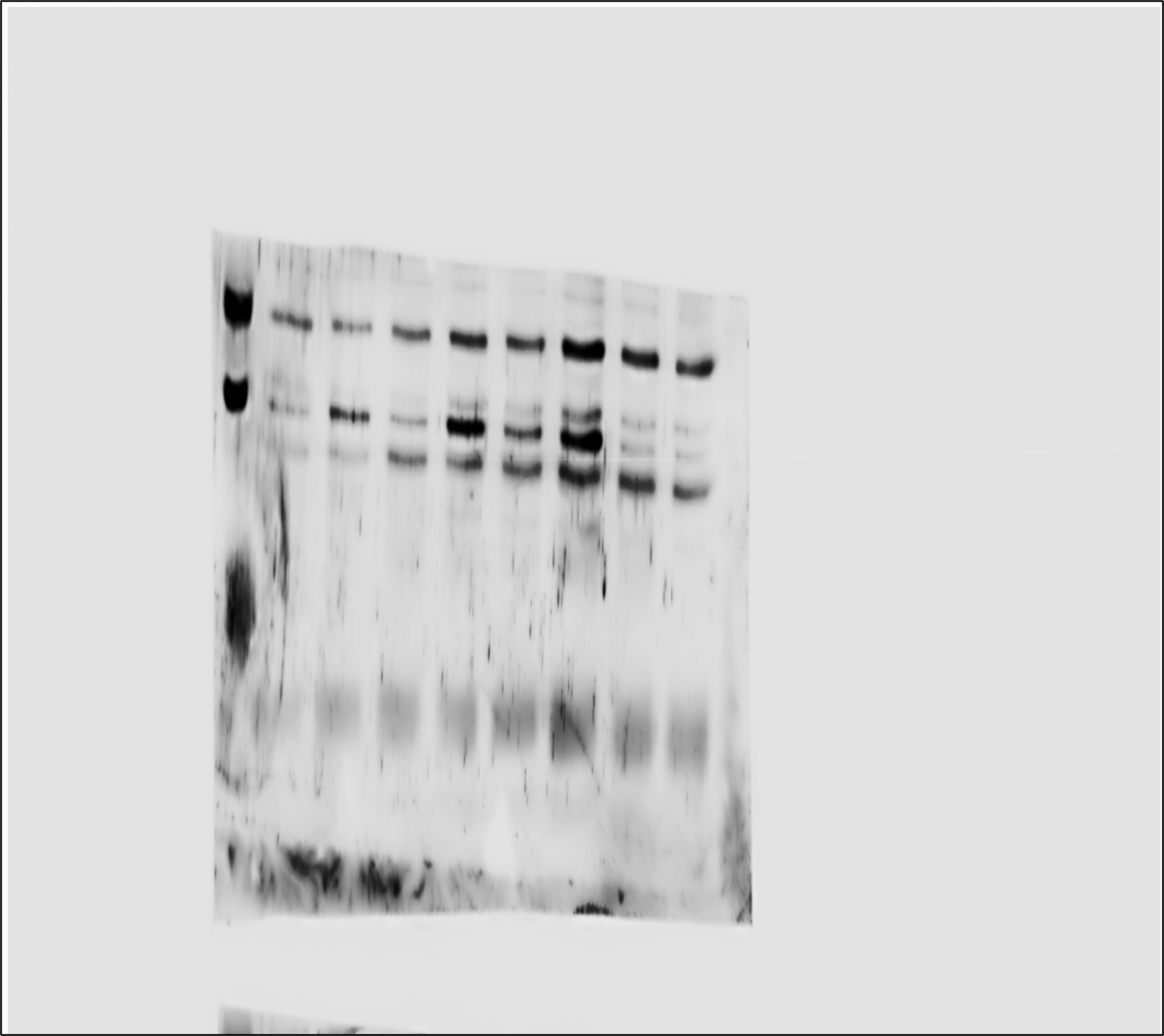

Supplement: Source data 1. — Figure 2. Uncropped, annotated immunoblot (Source data 1) and raw image files (Source data 2, 3) for DHFR and FtsZ in various E. coli mutants. Specific bands were identified based on molecular weight and absence from lysate from E. coli ΔfolA. Figure 2D. Uncropped, annotated immunoblot (Source data 1) and raw image files (Source data 2, 3, 4) for DHFR and FtsZ in various trimethoprim resistant E. coli mutants. Specific bands were identified based on molecular weight and absence from lysate from E. coli ΔfolA. Figure 2E. Uncropped, annotated immunoblot (Source data 1) and raw image files (Source data 2, 3, 4) for DHFR and FtsZ in various trimethoprim resistant E. coli mutants (TMPR1-5) and their ΔphoP derivatives. Specific bands were identified based on molecular weight and absence from lysate from E. coli ΔfolA. Figure 3E. Uncropped, annotated immunoblot (Source data 1) and raw image files (Source data 2, 3) for plasmid-expressed His-tagged DHFR or its mutant alleles in E. coli in the presence of indicated concentrations of inducer (IPTG). Figure 2—figure supplement 3. Uncropped, annotated immunoblot (Source data 1) and raw image files (Source data 2, 3) for DHFR and FtsZ in indicated E. coli mutants. Specific bands were identified based on molecular weight and absence from lysate from E. coli ΔfolA. [file elife-70931-supp4.zip › Source data-revised/Figure 2E-source data 2.tif]

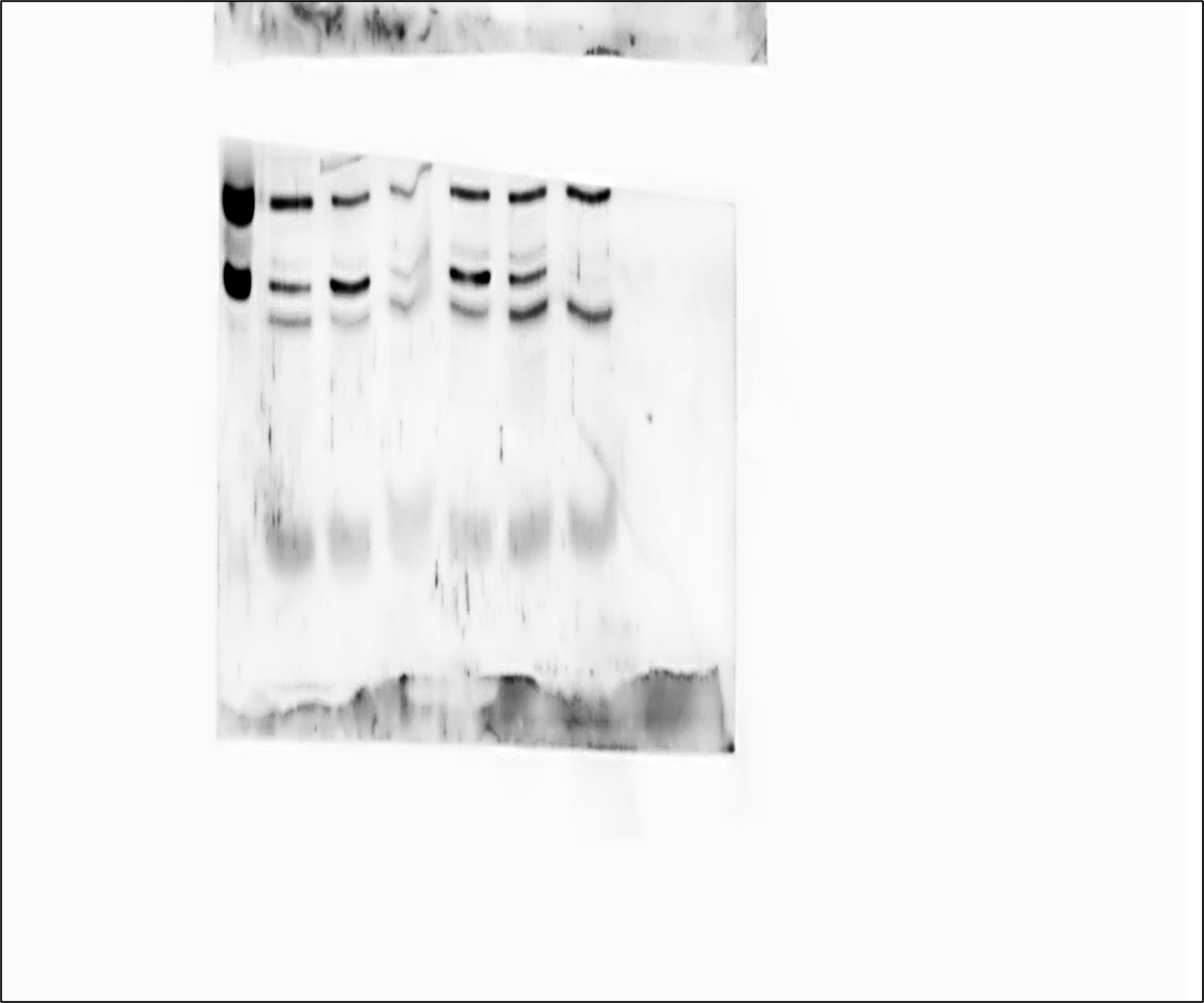

Supplement: Source data 1. — Figure 2. Uncropped, annotated immunoblot (Source data 1) and raw image files (Source data 2, 3) for DHFR and FtsZ in various E. coli mutants. Specific bands were identified based on molecular weight and absence from lysate from E. coli ΔfolA. Figure 2D. Uncropped, annotated immunoblot (Source data 1) and raw image files (Source data 2, 3, 4) for DHFR and FtsZ in various trimethoprim resistant E. coli mutants. Specific bands were identified based on molecular weight and absence from lysate from E. coli ΔfolA. Figure 2E. Uncropped, annotated immunoblot (Source data 1) and raw image files (Source data 2, 3, 4) for DHFR and FtsZ in various trimethoprim resistant E. coli mutants (TMPR1-5) and their ΔphoP derivatives. Specific bands were identified based on molecular weight and absence from lysate from E. coli ΔfolA. Figure 3E. Uncropped, annotated immunoblot (Source data 1) and raw image files (Source data 2, 3) for plasmid-expressed His-tagged DHFR or its mutant alleles in E. coli in the presence of indicated concentrations of inducer (IPTG). Figure 2—figure supplement 3. Uncropped, annotated immunoblot (Source data 1) and raw image files (Source data 2, 3) for DHFR and FtsZ in indicated E. coli mutants. Specific bands were identified based on molecular weight and absence from lysate from E. coli ΔfolA. [file elife-70931-supp4.zip › Source data-revised/Figure 2E-source data 3.tif]

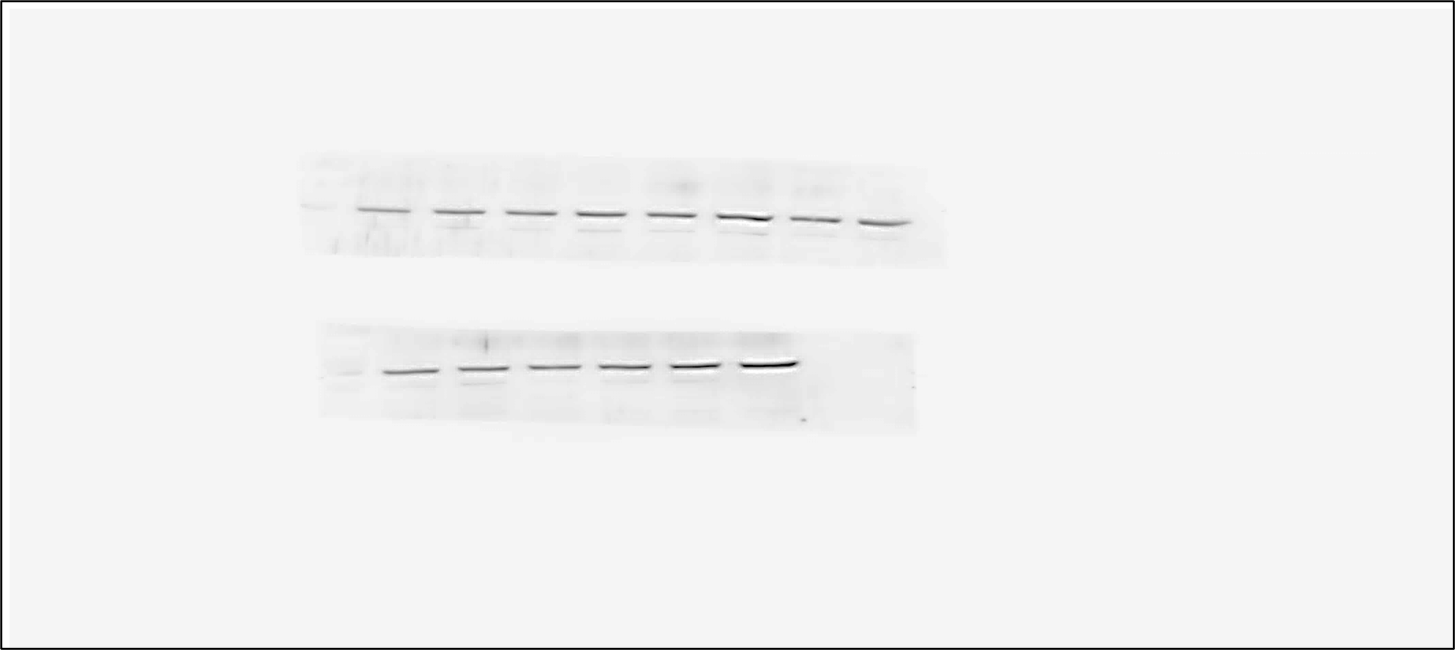

Supplement: Source data 1. — Figure 2. Uncropped, annotated immunoblot (Source data 1) and raw image files (Source data 2, 3) for DHFR and FtsZ in various E. coli mutants. Specific bands were identified based on molecular weight and absence from lysate from E. coli ΔfolA. Figure 2D. Uncropped, annotated immunoblot (Source data 1) and raw image files (Source data 2, 3, 4) for DHFR and FtsZ in various trimethoprim resistant E. coli mutants. Specific bands were identified based on molecular weight and absence from lysate from E. coli ΔfolA. Figure 2E. Uncropped, annotated immunoblot (Source data 1) and raw image files (Source data 2, 3, 4) for DHFR and FtsZ in various trimethoprim resistant E. coli mutants (TMPR1-5) and their ΔphoP derivatives. Specific bands were identified based on molecular weight and absence from lysate from E. coli ΔfolA. Figure 3E. Uncropped, annotated immunoblot (Source data 1) and raw image files (Source data 2, 3) for plasmid-expressed His-tagged DHFR or its mutant alleles in E. coli in the presence of indicated concentrations of inducer (IPTG). Figure 2—figure supplement 3. Uncropped, annotated immunoblot (Source data 1) and raw image files (Source data 2, 3) for DHFR and FtsZ in indicated E. coli mutants. Specific bands were identified based on molecular weight and absence from lysate from E. coli ΔfolA. [file elife-70931-supp4.zip › Source data-revised/Figure 2E-source data 4.tif]

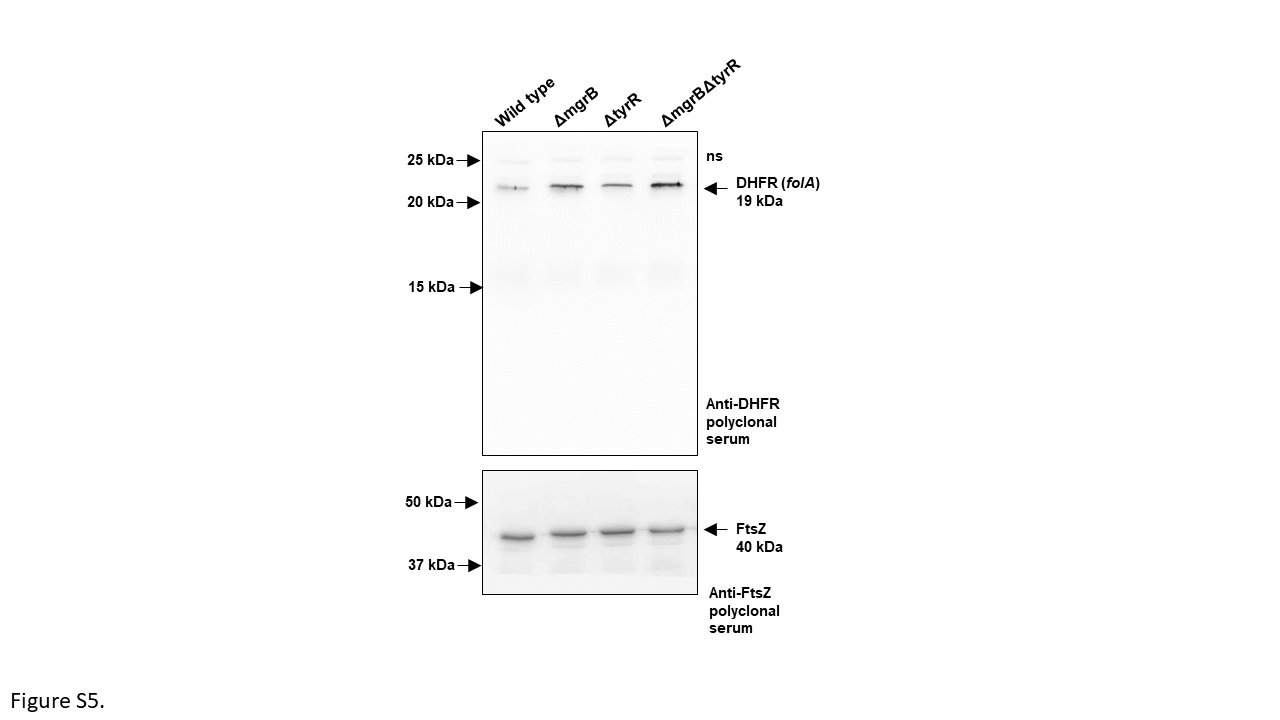

Supplement: Source data 1. — Figure 2. Uncropped, annotated immunoblot (Source data 1) and raw image files (Source data 2, 3) for DHFR and FtsZ in various E. coli mutants. Specific bands were identified based on molecular weight and absence from lysate from E. coli ΔfolA. Figure 2D. Uncropped, annotated immunoblot (Source data 1) and raw image files (Source data 2, 3, 4) for DHFR and FtsZ in various trimethoprim resistant E. coli mutants. Specific bands were identified based on molecular weight and absence from lysate from E. coli ΔfolA. Figure 2E. Uncropped, annotated immunoblot (Source data 1) and raw image files (Source data 2, 3, 4) for DHFR and FtsZ in various trimethoprim resistant E. coli mutants (TMPR1-5) and their ΔphoP derivatives. Specific bands were identified based on molecular weight and absence from lysate from E. coli ΔfolA. Figure 3E. Uncropped, annotated immunoblot (Source data 1) and raw image files (Source data 2, 3) for plasmid-expressed His-tagged DHFR or its mutant alleles in E. coli in the presence of indicated concentrations of inducer (IPTG). Figure 2—figure supplement 3. Uncropped, annotated immunoblot (Source data 1) and raw image files (Source data 2, 3) for DHFR and FtsZ in indicated E. coli mutants. Specific bands were identified based on molecular weight and absence from lysate from E. coli ΔfolA. [file elife-70931-supp4.zip › Source data-revised/Figure 2-figure supplement 3-source data 1.tif]

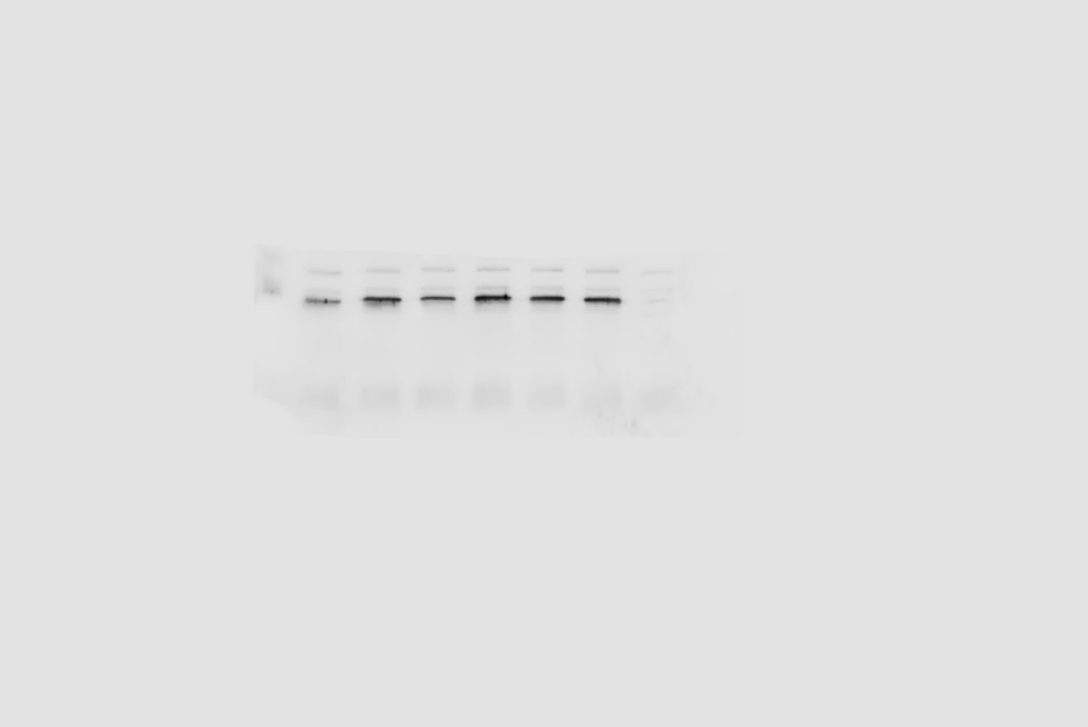

Supplement: Source data 1. — Figure 2. Uncropped, annotated immunoblot (Source data 1) and raw image files (Source data 2, 3) for DHFR and FtsZ in various E. coli mutants. Specific bands were identified based on molecular weight and absence from lysate from E. coli ΔfolA. Figure 2D. Uncropped, annotated immunoblot (Source data 1) and raw image files (Source data 2, 3, 4) for DHFR and FtsZ in various trimethoprim resistant E. coli mutants. Specific bands were identified based on molecular weight and absence from lysate from E. coli ΔfolA. Figure 2E. Uncropped, annotated immunoblot (Source data 1) and raw image files (Source data 2, 3, 4) for DHFR and FtsZ in various trimethoprim resistant E. coli mutants (TMPR1-5) and their ΔphoP derivatives. Specific bands were identified based on molecular weight and absence from lysate from E. coli ΔfolA. Figure 3E. Uncropped, annotated immunoblot (Source data 1) and raw image files (Source data 2, 3) for plasmid-expressed His-tagged DHFR or its mutant alleles in E. coli in the presence of indicated concentrations of inducer (IPTG). Figure 2—figure supplement 3. Uncropped, annotated immunoblot (Source data 1) and raw image files (Source data 2, 3) for DHFR and FtsZ in indicated E. coli mutants. Specific bands were identified based on molecular weight and absence from lysate from E. coli ΔfolA. [file elife-70931-supp4.zip › Source data-revised/Figure 2-figure supplement 3-source data 2.tif]

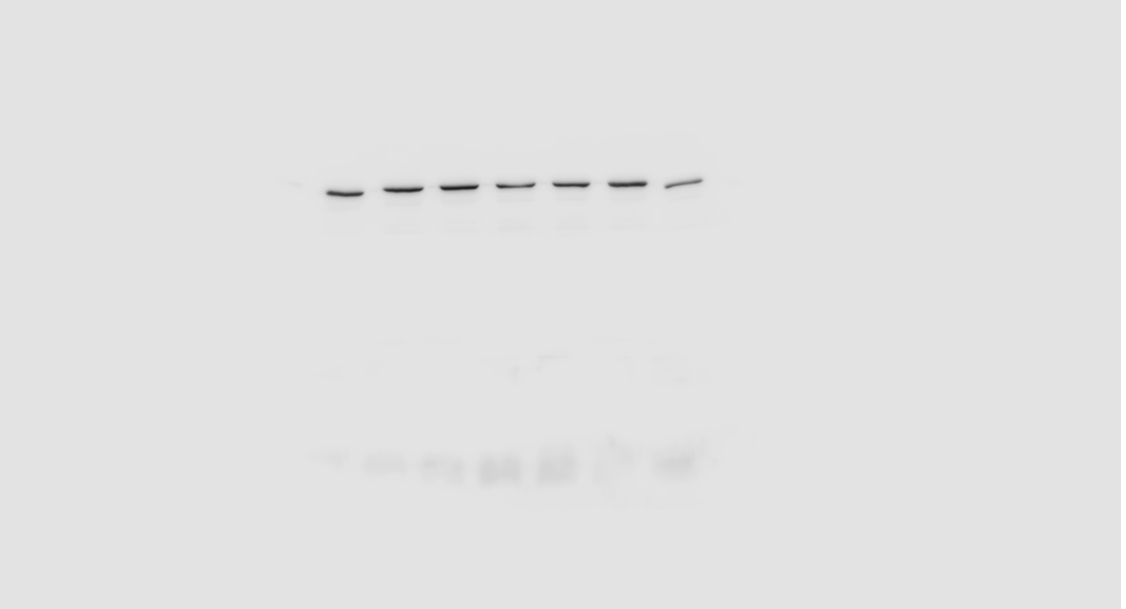

Supplement: Source data 1. — Figure 2. Uncropped, annotated immunoblot (Source data 1) and raw image files (Source data 2, 3) for DHFR and FtsZ in various E. coli mutants. Specific bands were identified based on molecular weight and absence from lysate from E. coli ΔfolA. Figure 2D. Uncropped, annotated immunoblot (Source data 1) and raw image files (Source data 2, 3, 4) for DHFR and FtsZ in various trimethoprim resistant E. coli mutants. Specific bands were identified based on molecular weight and absence from lysate from E. coli ΔfolA. Figure 2E. Uncropped, annotated immunoblot (Source data 1) and raw image files (Source data 2, 3, 4) for DHFR and FtsZ in various trimethoprim resistant E. coli mutants (TMPR1-5) and their ΔphoP derivatives. Specific bands were identified based on molecular weight and absence from lysate from E. coli ΔfolA. Figure 3E. Uncropped, annotated immunoblot (Source data 1) and raw image files (Source data 2, 3) for plasmid-expressed His-tagged DHFR or its mutant alleles in E. coli in the presence of indicated concentrations of inducer (IPTG). Figure 2—figure supplement 3. Uncropped, annotated immunoblot (Source data 1) and raw image files (Source data 2, 3) for DHFR and FtsZ in indicated E. coli mutants. Specific bands were identified based on molecular weight and absence from lysate from E. coli ΔfolA. [file elife-70931-supp4.zip › Source data-revised/Figure 2-figure supplement 3-source data 3.tif]

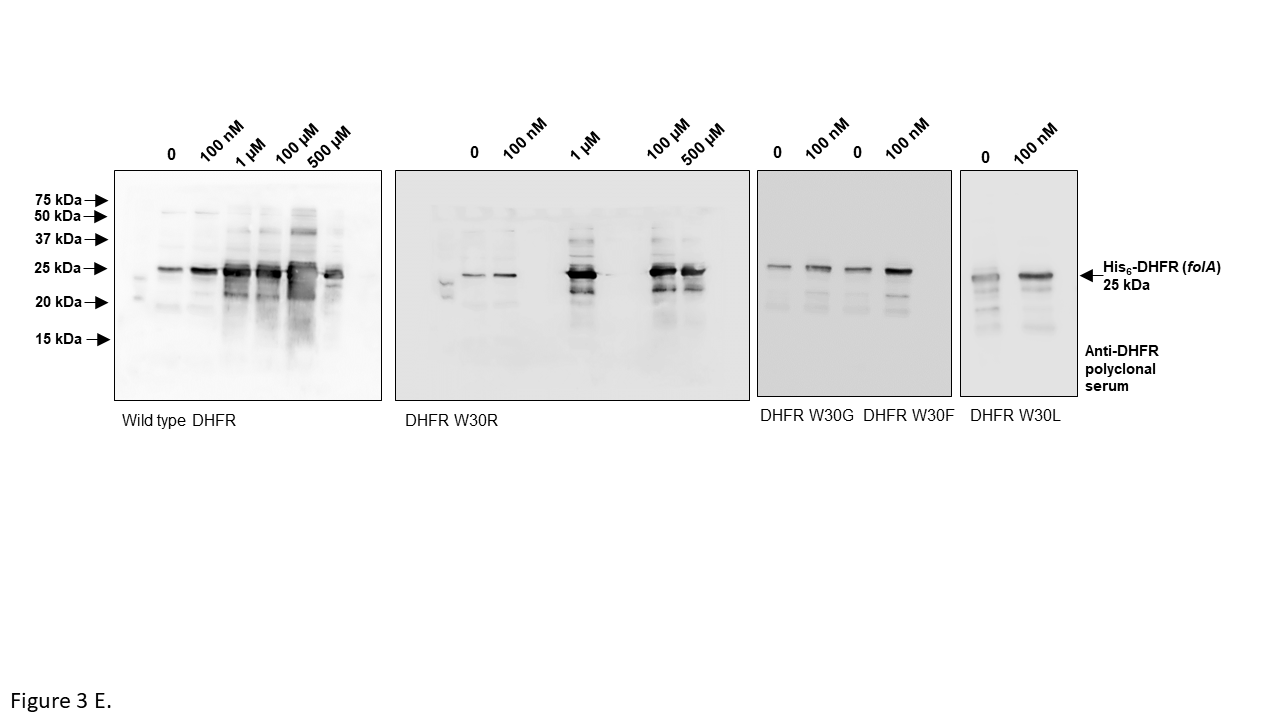

Supplement: Source data 1. — Figure 2. Uncropped, annotated immunoblot (Source data 1) and raw image files (Source data 2, 3) for DHFR and FtsZ in various E. coli mutants. Specific bands were identified based on molecular weight and absence from lysate from E. coli ΔfolA. Figure 2D. Uncropped, annotated immunoblot (Source data 1) and raw image files (Source data 2, 3, 4) for DHFR and FtsZ in various trimethoprim resistant E. coli mutants. Specific bands were identified based on molecular weight and absence from lysate from E. coli ΔfolA. Figure 2E. Uncropped, annotated immunoblot (Source data 1) and raw image files (Source data 2, 3, 4) for DHFR and FtsZ in various trimethoprim resistant E. coli mutants (TMPR1-5) and their ΔphoP derivatives. Specific bands were identified based on molecular weight and absence from lysate from E. coli ΔfolA. Figure 3E. Uncropped, annotated immunoblot (Source data 1) and raw image files (Source data 2, 3) for plasmid-expressed His-tagged DHFR or its mutant alleles in E. coli in the presence of indicated concentrations of inducer (IPTG). Figure 2—figure supplement 3. Uncropped, annotated immunoblot (Source data 1) and raw image files (Source data 2, 3) for DHFR and FtsZ in indicated E. coli mutants. Specific bands were identified based on molecular weight and absence from lysate from E. coli ΔfolA. [file elife-70931-supp4.zip › Source data-revised/Figure 3E-source data 1.tif]

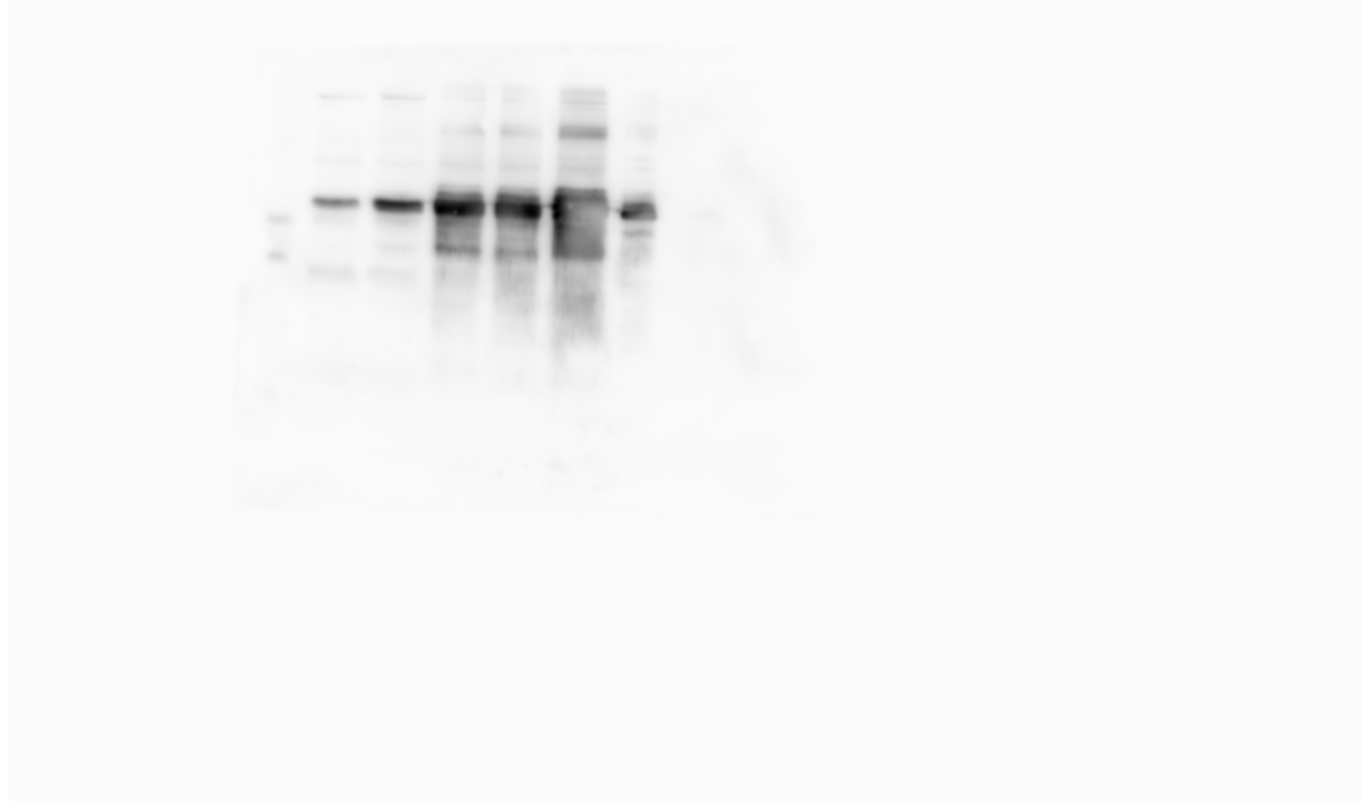

Supplement: Source data 1. — Figure 2. Uncropped, annotated immunoblot (Source data 1) and raw image files (Source data 2, 3) for DHFR and FtsZ in various E. coli mutants. Specific bands were identified based on molecular weight and absence from lysate from E. coli ΔfolA. Figure 2D. Uncropped, annotated immunoblot (Source data 1) and raw image files (Source data 2, 3, 4) for DHFR and FtsZ in various trimethoprim resistant E. coli mutants. Specific bands were identified based on molecular weight and absence from lysate from E. coli ΔfolA. Figure 2E. Uncropped, annotated immunoblot (Source data 1) and raw image files (Source data 2, 3, 4) for DHFR and FtsZ in various trimethoprim resistant E. coli mutants (TMPR1-5) and their ΔphoP derivatives. Specific bands were identified based on molecular weight and absence from lysate from E. coli ΔfolA. Figure 3E. Uncropped, annotated immunoblot (Source data 1) and raw image files (Source data 2, 3) for plasmid-expressed His-tagged DHFR or its mutant alleles in E. coli in the presence of indicated concentrations of inducer (IPTG). Figure 2—figure supplement 3. Uncropped, annotated immunoblot (Source data 1) and raw image files (Source data 2, 3) for DHFR and FtsZ in indicated E. coli mutants. Specific bands were identified based on molecular weight and absence from lysate from E. coli ΔfolA. [file elife-70931-supp4.zip › Source data-revised/Figure 3E-source data 2.tif]

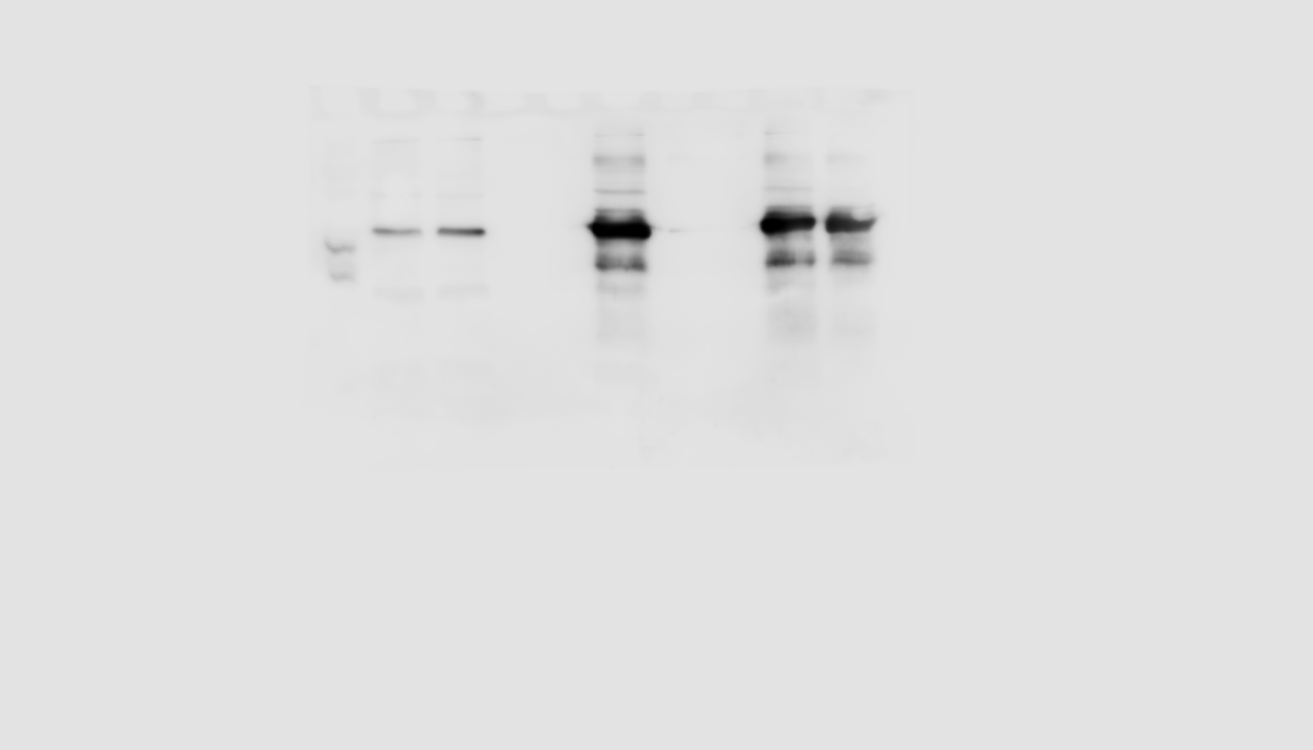

Supplement: Source data 1. — Figure 2. Uncropped, annotated immunoblot (Source data 1) and raw image files (Source data 2, 3) for DHFR and FtsZ in various E. coli mutants. Specific bands were identified based on molecular weight and absence from lysate from E. coli ΔfolA. Figure 2D. Uncropped, annotated immunoblot (Source data 1) and raw image files (Source data 2, 3, 4) for DHFR and FtsZ in various trimethoprim resistant E. coli mutants. Specific bands were identified based on molecular weight and absence from lysate from E. coli ΔfolA. Figure 2E. Uncropped, annotated immunoblot (Source data 1) and raw image files (Source data 2, 3, 4) for DHFR and FtsZ in various trimethoprim resistant E. coli mutants (TMPR1-5) and their ΔphoP derivatives. Specific bands were identified based on molecular weight and absence from lysate from E. coli ΔfolA. Figure 3E. Uncropped, annotated immunoblot (Source data 1) and raw image files (Source data 2, 3) for plasmid-expressed His-tagged DHFR or its mutant alleles in E. coli in the presence of indicated concentrations of inducer (IPTG). Figure 2—figure supplement 3. Uncropped, annotated immunoblot (Source data 1) and raw image files (Source data 2, 3) for DHFR and FtsZ in indicated E. coli mutants. Specific bands were identified based on molecular weight and absence from lysate from E. coli ΔfolA. [file elife-70931-supp4.zip › Source data-revised/Figure 3E-source data 3.tif]

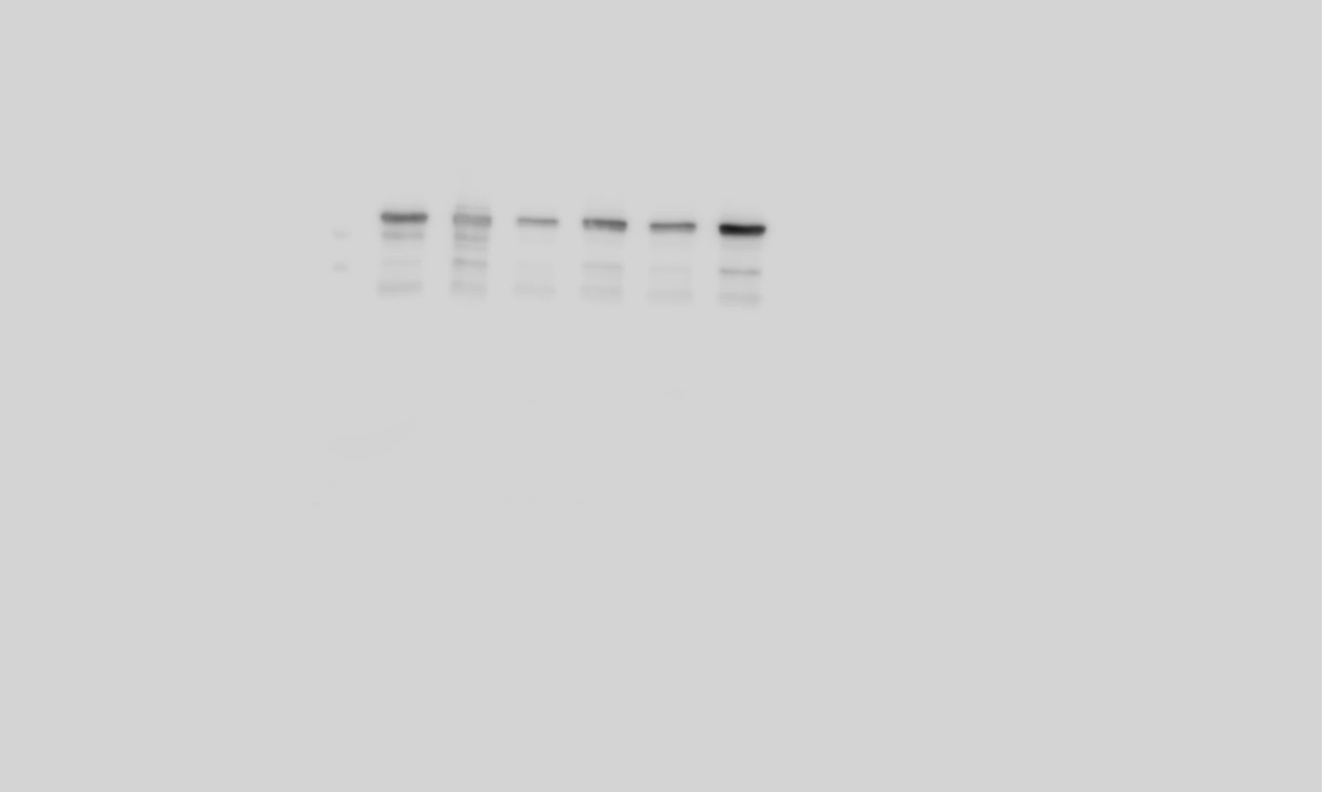

Supplement: Source data 1. — Figure 2. Uncropped, annotated immunoblot (Source data 1) and raw image files (Source data 2, 3) for DHFR and FtsZ in various E. coli mutants. Specific bands were identified based on molecular weight and absence from lysate from E. coli ΔfolA. Figure 2D. Uncropped, annotated immunoblot (Source data 1) and raw image files (Source data 2, 3, 4) for DHFR and FtsZ in various trimethoprim resistant E. coli mutants. Specific bands were identified based on molecular weight and absence from lysate from E. coli ΔfolA. Figure 2E. Uncropped, annotated immunoblot (Source data 1) and raw image files (Source data 2, 3, 4) for DHFR and FtsZ in various trimethoprim resistant E. coli mutants (TMPR1-5) and their ΔphoP derivatives. Specific bands were identified based on molecular weight and absence from lysate from E. coli ΔfolA. Figure 3E. Uncropped, annotated immunoblot (Source data 1) and raw image files (Source data 2, 3) for plasmid-expressed His-tagged DHFR or its mutant alleles in E. coli in the presence of indicated concentrations of inducer (IPTG). Figure 2—figure supplement 3. Uncropped, annotated immunoblot (Source data 1) and raw image files (Source data 2, 3) for DHFR and FtsZ in indicated E. coli mutants. Specific bands were identified based on molecular weight and absence from lysate from E. coli ΔfolA. [file elife-70931-supp4.zip › Source data-revised/Figure 3E-source data 4.tif]
